# Using bispectral index and cerebral oximetry to guide hemodynamic therapy in high-risk surgical patients

**DOI:** 10.1186/2047-0525-2-11

**Published:** 2013-05-19

**Authors:** Heena Bidd, Audrey Tan, David Green

**Affiliations:** 1 King’s College Hospital NHS Foundation Trust, Denmark Hill, London, SE5 9RS, UK

**Keywords:** BIS, Depth of anesthesia, Cardiac output monitoring, Cerebral oximetry, Triple low, LiDCOrapid, Intraoperative optimization, Goal-directed therapy

## Abstract

High-risk surgery represents 12.5% of cases but contributes 80% of deaths in the elderly population. Reduction in morbidity and mortality by the use of intervention strategies could result in thousands of lives being saved and savings of up to £400m per annum in the UK. This has resulted in the drive towards goal-directed therapy and intraoperative flow optimization of high-risk surgical patients being advocated by authorities such as the National Institute of Health and Care Excellence and the Association of Anaesthetists of Great Britain and Ireland.

Conventional intraoperative monitoring gives little insight into the profound physiological changes occurring as a result of anesthesia and surgery. The build-up of an oxygen debt is associated with a poor outcome and strategies have been developed in the postoperative period to improve outcomes by repayment of this debt. New monitoring technologies such as minimally invasive cardiac output, depth of anesthesia and cerebral oximetry can minimize oxygen debt build-up. This has the potential to reduce complications and lessen the need for postoperative optimization in high-dependency areas.

Flow monitoring has thus emerged as essential during intraoperative monitoring in high-risk surgery. However, evidence suggests that current optimization strategies of deliberately increasing flow to meet predefined targets may not reduce mortality.

Could the addition of depth of anesthesia and cerebral and tissue oximetry monitoring produce a further improvement in outcomes?

Retrospective studies indicate a combination of excessive depth of anesthesia hypotension and low anesthesia requirement results in increased mortality and length of hospital stay.

Near infrared technology allows assessment and maintenance of cerebral and tissue oxygenation, a strategy, which has been associated with improved outcomes. The suggestion that the brain is an index organ for tissue oxygenation, especially in the elderly, indicates a role for this technology in the intraoperative period to assess the adequacy of oxygen delivery and reduce the build-up of an oxygen debt.

The aim of this article is to make the case for depth of anesthesia and cerebral oximetry alongside flow monitoring as a strategy for reducing oxygen debt during high-risk surgery and further improve outcomes in high-risk surgical patients.

## Introduction

Intraoperative hemodynamic optimization in high-risk surgical patients is becoming a gold standard in anesthetic practice in the UK [1]. It is clear that the monitoring standards set by the Association of Anaesthetists of Great Britain and Ireland, that is, electrocardiography (ECG), pulse oximetry, end tidal carbon dioxide and non-invasive blood pressure, give little indication of the adequacy of oxygen delivery (DO_2_) to the patient during surgery [2]. The build-up of an oxygen debt during high-risk surgery, which can lead to an increase of postoperative complications and mortality, was considered inevitable [3,4]. Although repayment of the oxygen debt early in the postoperative period by goal-directed therapy (GDT) guided by sophisticated hemodynamic monitoring achieves beneficial outcomes [5,6], little emphasis has been placed on the potential role of maintaining DO_2_ intraoperatively and limiting build-up of the oxygen debt.

Pulse oximetry and monitoring end tidal carbon dioxide concentration, together with invasive arterial and central venous monitoring, continue to be the linchpin of conventional monitoring that most anesthetists use today even for high-risk patients. Advanced hemodynamic intervention strategies to manage these patients are evident in only around 10% of patients [7]. Based on the evidence that high-risk surgical procedures represent about 12.5% of the global volume of surgery carried out worldwide, this means that a realistic target of reduction in mortality from 10% in the control groups to 6% in the intervention groups could mean that potentially up to 800,000 lives could be saved by hemodynamic intervention strategies [8].

However, the situation may be changing as recent guidelines from the UK’s National Institute of Health and Care Excellence (NICE) have emphasized better outcomes and cost savings from hemodynamic monitoring by use of oesophageal Doppler monitoring (ODM) (Deltex Medical Group, Chichester, UK) [1,9]. This has now been extended to include all flow monitoring technologies with recent guidelines published on the management of proximal femoral fractures by the Association of Anaesthetists of Great Britain and Ireland recommending that we should consider hemodynamic monitoring, depth of anesthesia (DOA) monitoring and the use of cerebral oximetry [10]. A recent consensus statement from the Enhanced Recovery Partnership has also highlighted the importance of individualized goal- directed fluid therapy alongside the use of hemodynamic monitoring stating that ‘all Anaesthetists caring for patients undergoing intermediate or major surgery should have cardiac output measuring technologies immediately available and be trained to use them’ [11]. NICE has now extended their guidance to include a recommendation for the use of depth of anesthesia monitoring in these high-risk (elderly) patients [12].

### What about the role of combined monitoring?

In a review published in 2010 it was proposed that hemodynamic monitors combined with the use of depth of anesthesia and cerebral oximetry monitoring might further improve outcomes. Although there is limited evidence on the use of combined monitoring, the case for its use will be made [13].

### Cerebral oximetry

It is possible to measure cerebral and tissue oxygenation (regional oxygen saturation, rSO_2_) by the use of near infrared technology (NIRS) [14]. The Invos cerebral and tissue oximeter (Invos CTO, Covidien, CO, USA) was the first available for routine clinical use. Although there are now four main manufacturers employing this technology, opinions are still divided as to its place in routine anesthesia practice [15]. Nevertheless, the cerebral and tissue oximeter has been proposed as providing an index of brain and tissue oxygenation. The proposition that the brain is an index organ [16] is based on the fact that significant falls in rSO_2_ predict poor outcomes (both cerebral and non-cerebral) in both cardiac [17,18] and non-cardiac surgical patients [19]. Significant rSO_2_ desaturation may occur in up to 30% of major non-cardiac surgical procedures in elderly patients [19] and is usually associated with blood loss [20]. More importantly randomized controlled studies suggest that maintenance of rSO_2_ within 10% to 20% of baseline in these same patient groups reduces complications [16,18]. Although the normal rSO_2_ in a fit young patient may be around 60% to 70%, representing a venous weighted measure of cerebral tissue oxygenation, values as low as 35% are sometimes seen in elderly patients presenting for surgery and may be a significant predictor of a poor outcome [21]. A recent study suggests that rSO_2_ is a potentially important ‘biomarker to measure in heart failure patients and suggests that it may be a useful marker of target organ perfusion’ [22]. What is most interesting is that this monitor provides an early warning system for picking up imbalances between cerebral oxygen supply and demand, specifically deficient cerebral oxygen delivery, and if placed over the forehead, acts as an indicator of ischemia in the watershed area of the brain – that area supplied by the middle and anterior cerebral arteries. Other monitors may not identify these abnormalities [16].

Thus, a pre-induction measure of rSO_2_ for an elective patient provides a starting point from which to measure cumulative oxygen desaturation and also acts as a predictor of the likelihood of intraoperative and postoperative complications.

### Propositions concerning rSO_2_ and oxygen debt

Oxygen debt can be assessed by measuring the difference in oxygen consumption (VO_2_) intraoperatively and comparing this value with that obtained in the immediate preoperative period [3]. However, calculation of oxygen consumption requires pulmonary artery catheterization to determine the mixed venous oxygen content as well as thermodilution to measure cardiac output (CO) and cannot easily be employed routinely. An association exists between cumulative oxygen debt occurring during surgery and the immediate postoperative period with a poor outcome [3,4]. These deleterious effects may be ameliorated by artificially increasing oxygen delivery to so-called goal-directed therapy (GDT) targets of 600 ml.min-^1^.m^-2^ using inotropes and fluids in the immediate postoperative period [23]. However, such aggressive therapy may not be necessary if oxygen delivery can be maintained and oxygen debt minimized intraoperatively. The Shoemaker studies indicated that the build-up of oxygen debt was usually associated with reduced oxygen delivery intraoperatively. Thus maintaining oxygen delivery seems to be a logical strategy for the elective patient in order to minimize the build-up of an oxygen debt. In this context, the cerebral and tissue oximeter is arguably the best continuous non-invasive assessment of the adequacy of cerebral and tissue oxygenation, especially in elderly, elective surgical patients [14]. If oxygen delivery is insufficient to supply enough oxygen for adequate cellular activity then rSO_2_ will fall (signifying increased oxygen extraction when supply is deficient) and should be corrected. A recent review also suggests that maintaining tissue oxygenation may improve outcomes [14].

## Depth of anesthesia monitoring

### Awareness

Recent prospective, double-blinded studies have produced conflicting data on whether or not bispectral index (BIS) monitoring reduces patient awareness compared with other techniques such as close monitoring of end tidal anesthetic concentration in (say) the 0.7 to 1.3 minimal alveolar concentration (MAC) range. However, the trials are plagued with discrepancies in their protocols that render some of the results meaningless [24-26]. Nevertheless, what is clear is that patients who consistently have high BIS numbers are the ones most likely to be aware [27].

### Too deep?

The recommended optimal ranges of maintenance BIS values are between 40 and 60 [28]. Some evidence points to an association of excessive depth of anesthesia with poor outcomes especially in high-risk patients [29-31]. However, not all the data supports this hypothesis [32,33]. Two studies carried out by analyzing retrospective data of two classes of patients who participated in the B-unaware study [26] showed conflicting results. A persistent BIS value below 45 led to a poor outcome in the cardiac surgical cohort [30]. However, when this analysis was repeated for the non-cardiac surgical patients, no difference in outcomes was found [33]. The proposed explanation for this paradox was ‘that BIS values lower than 45 are likely markers of systemic illness, poor cardiac function, or complicated intraoperative course,’ which explains the difference in outcomes for cardiac versus non-cardiac surgical patients [33].

**Figure 1:**
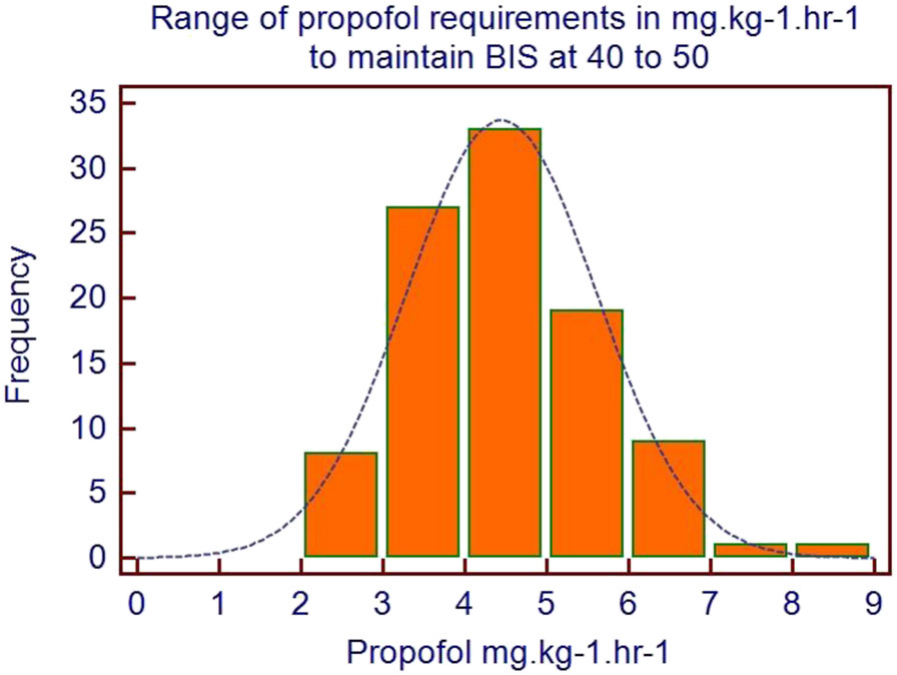
**Range of propofol requirements in mg.kg**^**-1**^**.hr**^**-1 **^**to maintain BIS in the 40 to 60 range.** Note the nearly fivefold variation in propofol requirements. BIS: bispectral index.

**Figure 2:**
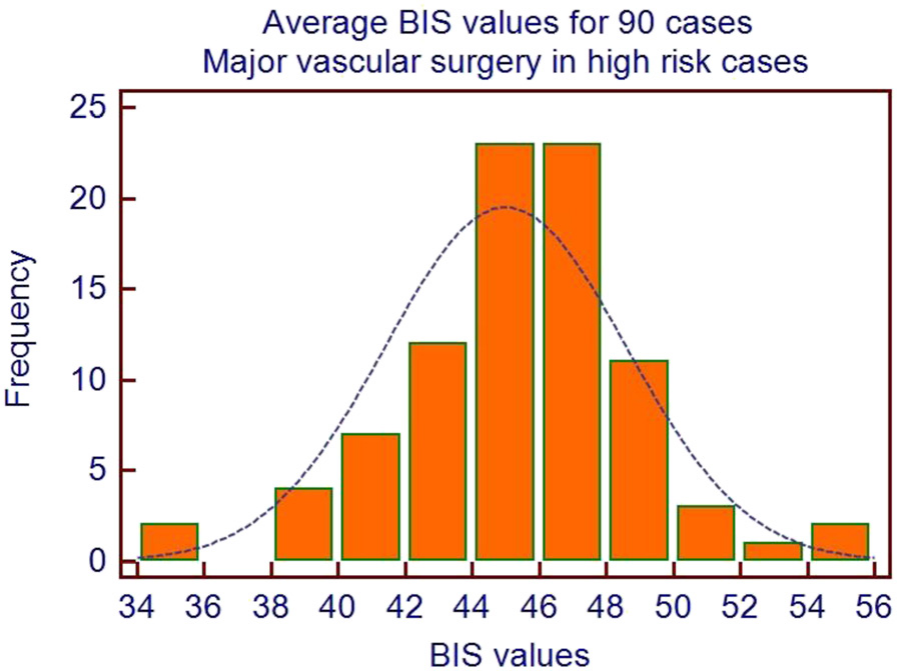
**BIS values obtained for a series of 90 high-risk patients undergoing major vascular surgery using total intravenous anesthesia with propofol and remifentanil.** The average BIS was 45 and only 6 patients had BIS maintained below the normal lower range of 40. BIS: bispectral index.

This explanation must be vigorously challenged, as it seems naïve to assume that all patients have the same pharmaco-genetic make-up and if BIS is low then the concentration of anesthetic should be reduced. Our own data (Figure 1) show a fivefold variation in propofol requirements to maintain BIS in the 40 to 60 range (Figure 2) in a series of 103 high-risk, elderly patients. Without the correct use of depth of anesthesia monitoring it is clear that conventional population-based targets may lead to many patients being too deeply anesthetized whilst others will be too light.

Kertai *et al.*[33] also state that ‘putting these data together, if the pharmacologically paralyzed patient were hypotensive and anesthetic was delivered at a clinically acceptable concentration with BIS values less than 45 (e.g. BIS of 39) the appropriate initial intervention might not be to decrease anesthesia. Instead, it might be preferable to treat hypotension with fluids, for example, or a drug such as norepinephrine or phenylephrine.’ Surely, if the patient were hypotensive with a low BIS (say 29, but you would need a BIS monitor to know this), would it not be more appropriate to reduce the depth of anesthesia first, rather than give more fluids and vasopressors? It is also common to have hypotensive elderly patients with a high BIS value and here we firstly need to vigorously treat the causes of hypotension and then deepen the anesthetic. How can we do that rationally without hemodynamic and BIS monitoring? A recent study supports a very close relationship between stroke volume and BIS level, which confirms depth of anesthesia as a potent cause of reduced cardiac output and oxygen delivery presumably resulting from propofol-induced venous tone reduction [34]. Hence, for an elderly high-risk patient this could lead to a decrease in DO_2_ and even a build-up of oxygen debt [35]. A conflict of rational management occurs here as, although a recent recommendation by expert authors suggests that depth of anesthesia monitoring should be used for total intravenous anesthesia, the same authors also advise that the concentration of anesthetic should not be adjusted to keep BIS within the target range [36].

Two recent reviews of the evidence for the effect of depth of anesthesia on outcomes call for large-scale prospective, randomized controlled trials to be urgently carried out to support or contradict the hypothesis that low BIS is associated with poor outcomes [37,38]. Looking specifically at the incidence of delirium and cognition in the postoperative period in elderly patients, it would appear from the CODA trial that maintaining BIS in the 40 to 60 range significantly reduces the incidence of both these complications [39].

### Triple low

**Figure 3:**
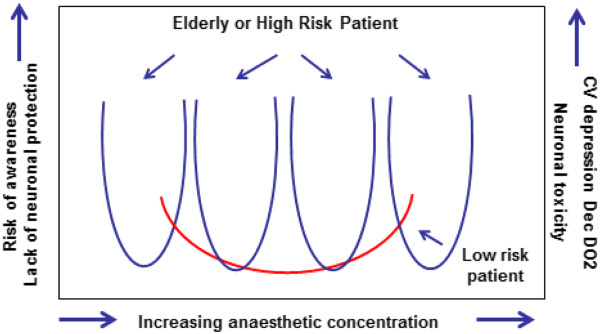
**Possible mechanism of how anesthetic concentration may affect outcomes.** Anesthetic concentration increase along the *x* axis. If the concentration is too low then there is a risk of awareness and lack of neuronal protection (left *y* axis). If the concentration is too high then there is a risk of cardiovascular depression with hypotension and decreased oxygen delivery (DO_2_) and a risk of neuronal toxicity (right *y* axis). The red arc indicates a low-risk patient where the risk of problems is small despite big changes in anesthetic concentration. The blue curves indicate the various likely responses of a high-risk patient where the response to anesthesia is, and the implications for getting it wrong, are much greater as indicated by the much steeper dose–response curves. CV: cardiovascular; DO_2_: oxygen delivery.

Retrospective analysis of a large anesthesia database at the Cleveland Clinic indicates an association between the cumulative time of being in a triple low state (low BIS <45, low minimal alveolar concentration, MAC < 0.8, and low mean arterial pressure, MAP < 75 mmHg) with a fourfold increase in 30-day mortality and increased length of hospital stay [40]. The study emphasizes that these values are referenced on patients whose mean BIS, MAP and MAC values are outside one SD of their population mean values and not necessarily outside the range normally tolerated as clinically acceptable by most anesthetists. Interestingly, low BIS on its own was not a predictor of poor outcomes in this series, although in a high-risk patient a low BIS might be associated with low MAP and low MAC. Low MAP and BIS values in those receiving low anesthetic MAC may identify patients who are ‘unusually sensitive to anesthesia and at risk for complications’ confirmed. Their suggestion that ‘Inadequate cerebral perfusion is perhaps the most interesting putative cause of low BIS because it is potentially amenable to hemodynamic intervention, such as giving vasopressors or fluids to improve MAP and brain perfusion’ highlights the potential role of cerebral oximetry and flow monitoring alongside BIS in these patients [40]. Figure 3 offers an explanation of how low BIS might be a potential cause of poor outcomes for high-risk/elderly patients but may be less predictive of outcomes in healthy patients. It should also be noted that although the emphasis has been on low BIS as a predictor of outcomes, a high BIS level may not only lead to awareness but also result in a lack of neuronal protection and lead to postoperative cognitive dysfunction [41], itself a cause of poor outcome [42].

### Hemodynamic monitoring

It is usual anesthetic practice to maintain a patient’s MAP within 20% of the patient’s baseline value. A common response to a fall in MAP is to administer fluid, either crystalloids such as lactated Ringer’s or colloids such as gelatins or starch. Recent studies, however, have suggested that some patients receive too much fluid during major surgery and a fluid restriction or zero-balance strategy may lead to better outcomes [43,44]. Most anesthetists still employ a maintenance fluid regime based originally on a paper published in 1961 by Shires *et al.*[45], who were the first group to propagate what is now seen by many as a myth, that is, third-space loss. They suggested up to 15 ml.kg^-1^.hr^-1^ of lactated Ringer’s be administered to replace the extracellular fluid that had been presumed lost from the functional extracellular fluid volume (FECV). This regime may result in a patient receiving up to an equivalent of 10 days normal Na^+^ requirement (700 mmol) in the space of a four-hour operation. Evidence exists that excess fluid may damage the glycocalyx and thus exacerbate loss of FECV [46]. This regimen may lead to poorer outcomes in comparison with GDT and restrictive regimens in colorectal surgery [47]. Despite this evidence, even the most recent trials using hemodynamic optimization protocols continue to presume this third-space loss, with fluid administered in the control group, and the intervention group, often amounting to more than 10 ml.kg^-1^.hr^-1^ of lactated Ringer’s or Hartmann’s solution [48,49]. A recent review of the evidence for third-space loss in surgery or following blood loss indicates that it does not occur [50] (see later).

### Oesophageal Doppler monitoring

**Figure 4:**
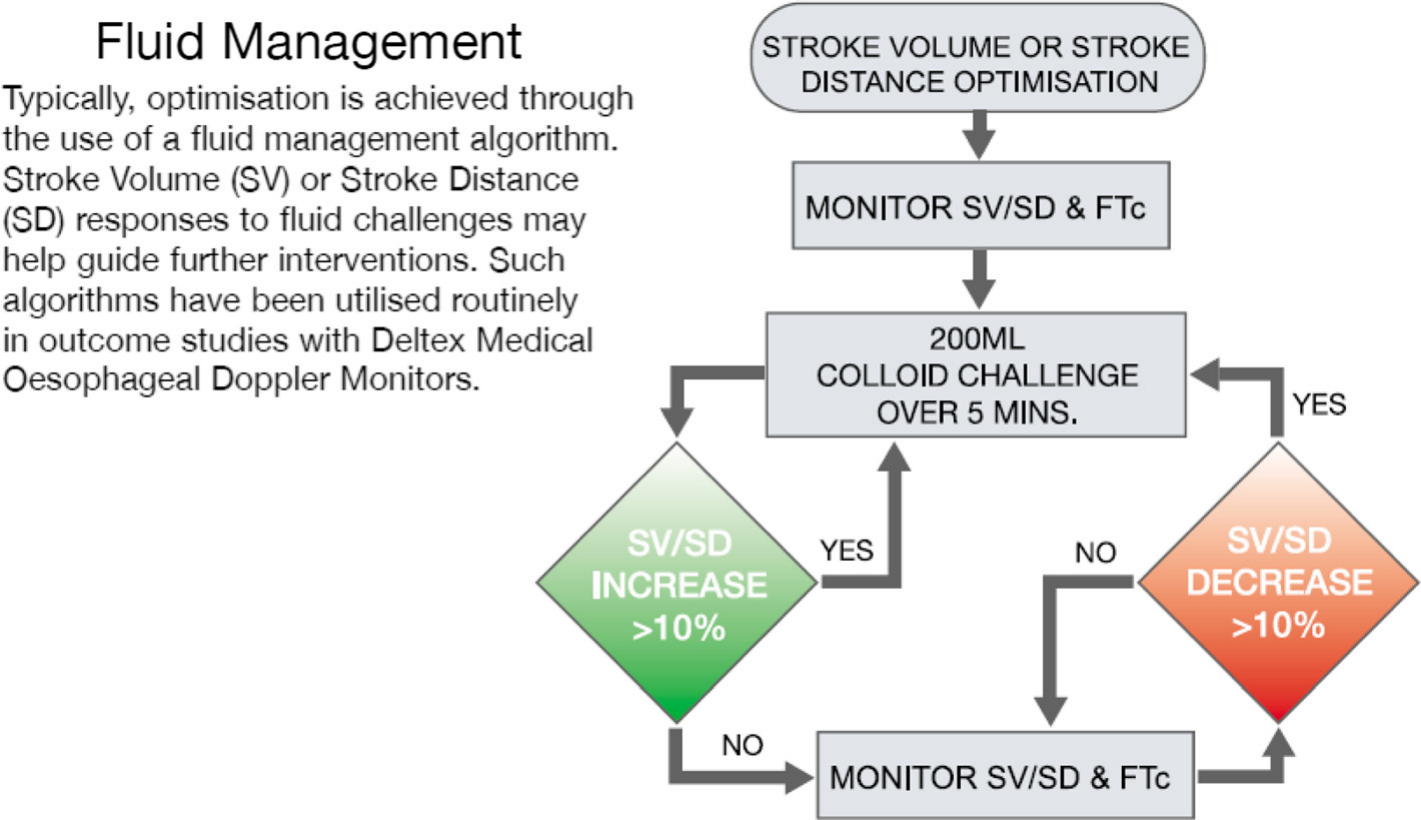
**Intraoperative fluid management strategy as proposed by the Deltex company (Deltex Medical, Chichester, UK) [**[55]**].** FTc: flow-time corrected; SD: stroke distance; SV: stroke volume.

The potential for intraoperative hemodynamic monitoring to improve outcome has mainly been derived from work on oesophageal Doppler monitoring [12-14]. Unfortunately the three main studies on which the NICE guidelines were based used different end points in assessing the adequacy of optimal hemodynamics. Gan *et al.*[51] and Noblett *et al.*[52] used optimization of the flow-time corrected value maintained between 350 and 400 ms whilst Wakeling *et al.*[53] used stroke volume maximization together with central venous pressure (CVP). The latter study led to the algorithm currently employed by Deltex (Deltex Medical PLC, Chichester, UK) the manufacturers of the Doppler monitor, which does not use CVP and flow-time corrected monitoring (Figure 4). When this algorithm was used in a recent study employing the ODM the intervention group actually had worse outcomes [48]. In addition, using a Doppler monitor to maximize stroke volume (SV) does not seem to produce any benefits in outcomes when compared with a restrictive, zero-balance technique, despite achieving higher nominal cardiac output in the oesophageal Doppler intervention group [54].

### Pulse contour and power analysis

There is surprisingly little data supporting the benefits of intraoperative (versus postoperative [23]) hemodynamic optimization using flow monitors, which analyze the arterial pressure waveform and convert it into flow. The LiDCOrapid monitor (LiDCO PLC, Cambridge, UK) analyses the whole arterial waveform and not just the systolic portion as do other devices and thus its estimates of stroke volume and cardiac output are less affected by changes in waveform morphology [56]. Despite some confusion in the literature [57], the algorithm used in the Rapid is the same as that used for the past 12 years in the LiDCOplus. The difference is that the latter is a calibrated value of the cardiac output whilst the Rapid uses the patient’s height, weight and age to scale the algorithm to produce a nominal cardiac output (nCO). This is then used as a reference pre-induction value in elective patients. Recent work suggests that this monitor is suitable for use for these high-risk patients [58,59]. The ability of such monitors to obtain a baseline value of the patient’s hemodynamic status prior to induction is an advantage and allows use of the patient as their own control, and the monitors are used to maintain nCO (and therefore oxygen delivery) close to this baseline value throughout surgery. Often there may be a fall in nCO post-induction of up to 50% in elderly patients with limited cardiac reserve [35]. It is clear that hemodynamic monitoring commencing post-induction may greatly underestimate the patient’s starting nCO and nDO_2_ values, making it impossible to use the patient as their own control. In a study by Lobo *et al.*[49], patients receiving goal-directed therapy to a target oxygen delivery of 600 ml.min^-1^.m^-2^ with dobutamine and fluids using the LiDCOplus with a restricted background infusion of LR of 4 ml.kg^-1^.hr^-1^ did better than those receiving the conventional regime of 12 ml.kg^-1^.hr^-1^ despite achieving lower goal-directed therapy targets [49].

### What about the role of conventional maintenance fluids given alongside hemodynamic optimization?

The definition of a maintenance fluid in the intraoperative period often confuses basic physiological losses (around 1 to 2 ml.kg^-1^.hr^-1^ of crystalloid and around 1 mmol.kg^-1^ of Na^+^ per day) with the excess loss due to third-space fluid loss (up to 15 ml.kg^-1^.hr^-1^ or 10 mmol.kg^-1^ Na^+^ per day). Since third-space loss during elective major surgery is now believed to be minimal [46,50,60] (see above), this strategy can result in considerable fluid overload and paradoxically may increase the risk of acute kidney injury in the perioperative period [61]. Elective patients are not usually fluid depleted; any fall in SV during the induction of anesthesia is likely due to alterations in venous capacitance and a fall in venous return and pre-load, making fluid an inappropriate choice of treatment in the early stages of anesthesia.

Any strategy that assumes that this loss still takes place is liable to result in fluid overload and a worsened outcome [48,49]. Using a stroke volume maximization strategy in patients in a background of excessive DOA again may result in excessive fluid administration. Thus, it would seem rational to base fluid requirement on hemodynamic monitor data with the patient at the optimum depth of anesthesia. In the recently completed *Optimise* trial the background maintenance fluid was restricted to 1.5ml.kg^-1^.hr^-1^[62]. Our own data suggest that optimal hemodynamic targets can be achieved with substantially reduced amounts of Na^+^ and fluid given (crystalloid maintenance), if hemodynamic monitors are used alongside BIS and a cerebral tissue oximeter [63].

### So, should we be employing a stroke volume maximization strategy?

The stroke volume maximization strategy is based on the false assumption that the Starling curve applies to an intact circulation and that the heart is a driver of tissue oxygenation. The mantra that maximizing CO and DO_2_ ‘must be good for the patient’ has no sound physiological basis and may be deleterious [46]. In fact, it is the tissues that regulate CO and not the other way round. So, it is probably unnecessary to try to maximize CO and DO_2_ during major surgery. Maintaining pre-induction DO_2_ in the intraoperative period seems a more logical strategy. Trying to drive DO_2_ to a target of 600 ml.min^-1^.m^-2^ in an elderly patient with a Hb of 10 g.dl^-1^ would necessitate a 50% increase in CO to achieve this end point and may impose unnecessary strain on the cardiovascular system. The ability of the tissues to regulate their own blood supply and DO_2_, especially the brain and muscle, was reviewed by Wolff [64]. A recent Cochrane review suggests that the benefits of a perioperative increase in global blood flow to explicitly defined goals and outcomes following surgery may have been exaggerated [65].

### How do we know that our oxygen delivery is adequate and how do we avoid a build-up of oxygen debt?

As mentioned earlier, it is very easy for oxygen debt to build up during major surgery and lead to postoperative complications. Although postoperative goal-directed therapy aimed at replacing oxygen debt is beneficial [23], it may not be required if the accumulation of an oxygen debt is kept to a minimum in the first instance. A logical strategy to maintain pre-induction DO_2_ in elective high-risk patients intraoperatively would be to use pre-induction monitoring. In our institution, for example, this is done by attaining the nCO using a LiDCOrapid monitor slaved off a radial arterial line before induction of anesthesia. During surgery nominal nCO and nominal DO_2_ (nDO_2_) are maintained within 10% of pre-induction values. This is achieved by the judicious use of vasopressors such as phenylephrine, commenced pre-induction, to maintain venous tone and venous return combined with fluid challenges, based on stroke volume variation (SVV) whilst ensuring optimal depth of anesthesia [66]. Maintenance of pre-induction values of rSO_2_ ensures, as best as we can, that oxygen supply/demand and tissue oxygenation are maintained, which may improve outcomes [14]. Falls in rSO_2_ are corrected by restoration of nCO, nDO_2_, MAP, oxygen saturation (SpO_2_) and ensuring normocapnia [19]. If rSO_2_ continues to fall then it is usually due to blood loss and anemia and is correctable by blood transfusion [20].

This strategy has given further insights into the problems arising when the blood volume is insufficient to fill the circulatory capacity due to a fall in venous tone. Any discrepancy between blood volume and circulatory capacity will mean there is a reduced blood volume on the arterial/capillary side of the circulation. With the increased capacity due to venodilation on induction there will be a reduction in venous return and thus SV and CO. Those organs with the most robust auto-regulatory capacity (heart and skeletal muscle) will sustain normal blood flow, whereas those with the least will be under-filled. The gut is the first to exhibit a defective blood flow, as suggested by gastric tonometry studies [67,68]. The next organ is probably the brain, though renal blood flow is also compromised with modest reductions in blood volume (or reduction in the relation of the blood volume to capacity). With careful responses to increased SVV (an indicator of excess venous capacity), there will be a resumption of blood flow to these most vulnerable organs. Some further work is needed to monitor the specific organs during assessment of volume status.

## Conclusion

Hemodynamic monitoring commenced pre-induction, together with maintenance of an adequate depth of anesthesia and cerebral oxygenation, helps to ensure intraoperative optimization of oxygen delivery. This not only minimizes or even eliminates any build-up of oxygen debt but also reduces fluid and sodium input, thus allowing true individualization of fluid therapy. Large-scale, prospective randomized trials are urgently needed to prove or disprove that this strategy improves patient outcomes [13].

## Abbreviations

BIS: Bispectral index; CO: Cardiac output; CV: Cardiovascular; CVP: Central venous pressure; DOA: Depth of anesthesia; DO2: Oxygen delivery; ECG: Electrocardiography; FECV: Functional extracellular fluid volume; FTc: Flow-time corrected; GDT: Goal-directed therapy; Hb: Haemoglobin; MAC: Minimal alveolar concentration; MAP: Mean arterial pressure; nCO: nominal cardiac output; nDO2: nominal oxygen delivery; NICE: National Institute for Health and Care Excellence; ODM: Oesophageal Doppler monitor; rSO2: Cerebral oxygenation; SD: Stroke distance; SpO2: Oxygen saturation; SV: Stroke volume; SVV: Stroke volume variation.

## Competing interests

DG has received monitoring equipment on loan and some disposables from Edwards Laboratories, LiDCO PLC, Philips Respironics, Deltex Medical Group and Covidien Inc. He has also received traveling expenses from LiDCO PLC to speak at meetings and he has received honoraria and traveling expenses from Glaxo Smith Kline PLC and Covidien Inc. to speak at meetings. Heena Bidd and Audrey Tan’s research resident posts at King’s College Hospital were partially funded with a £15k annual grant from LiDCO PLC.

## Authors’ contributions

All authors contributed equally to writing the review. All authors read and approved the final manuscript.
